# Nerve-Sparing Laparoscopic Radical Hysterectomy (nsLRH) without Adjuvant Therapy in FIGO Stage IB3 Cervical Cancer Patients: Surgical Technique and Survival Outcomes

**DOI:** 10.3390/cancers16193355

**Published:** 2024-09-30

**Authors:** Roberto Tozzi, Sofia Bigardi, Giulia Spagnol, Federico Ferrari, Carlo Saccardi, Marco Noventa, Matteo Marchetti

**Affiliations:** 1 Department of Gynecology and Obstetrics, Division of Women and Children’s Health, University of Padua, 35128 Padua, Italygiuliaspagnol.ts@gmail.com (G.S.); carlo.saccardi@unipd.it (C.S.); marco.noventa.2@unipd.it (M.N.); matteo.marchetti@unipd.it (M.M.); 2 Department of Clinical and Experimental Sciences, University of Brescia, 35122 Brescia, Italy

**Keywords:** cervical cancer IB3, nerve-sparing radical hysterectomy, laparoscopic radical hysterectomy, survival, morbidity

## Abstract

**Simple Summary:**

Since no randomized clinical trial has compared surgery to chemoradiotherapy (CTRT) in FIGO stage IB3 cervical cancer patients, the optimal management remains controversial. In our study, nerve-sparing laparoscopic radical hysterectomy (nsLRH) proved to be safe and effective. The encouraging results on toxicity and survival, when compared to CTRT, warrant further investigation and reinforce the idea that surgery is a valid option for these patients.

**Abstract:**

(1) Background: In 2018 FIGO reclassified tumors confined to the cervix larger than 4 cm as stage IB3. Although concurrent CTRT has been the standard of care and surgery the alternative, optimal management remains controversial due to the lack of direct comparison between surgery and CTRT. (2) Methods: This prospective observational study investigated the efficacy, safety and oncologic outcomes of nerve-sparing laparoscopic radical hysterectomy (nsLRH) for FIGO stage IB3 cervical cancer patients (IB3). From 2009 to 2023, IB3 patients underwent laparoscopic pelvic lymphadenectomies with frozen section analysis, followed by a nsLRH if the lymph nodes were tumor-free. No uterine manipulator was used and the vaginal cuff was sealed before retrieving the specimen. Intermediate-risk patients were under close observation without adjuvant therapy. Outcomes were monitored until 2023. (3) Results: During the study period, 74 IB3 patients were treated. Sixty-eight (91.9%) underwent a nsLRH. A complete resection with negative margins was achieved in all cases. At a median of 68 months of follow-up, the disease-free survival (DFS) rate was 89.7% and the overall survival (OS) rate was 93.1%. The overall complication rate was 23.5% and there were no grade 4–5 complications. (4) Conclusions: In patients with IB3 cervical cancer, a nsLRH is safe and effective. While awaiting the results from ongoing randomized trials, these findings support nsLRH as a viable treatment.

## 1. Introduction

In 2018, the International Federation of Gynecology and Obstetrics (FIGO) updated the staging of cervical cancer [1]. Patients with tumors confined to the cervix sized >4 cm, previously categorized as IB2, are now classified as FIGO stage IB3 cervical cancer (IB3).

Traditionally, the treatment for this stage of tumor has been radical surgery or radiotherapy (RT) [2]. Based on the results of several clinical trials, concurrent chemoradiation (CTRT) has fully replaced RT alone in the treatment of local advanced cervical cancer [3,4,5,6,7,8,9]. Although not based on published comparative data, there is an international consensus on unimodal treatment to avoid the combination of surgery and RT with its supposed increase in treatment morbidity [10,11]; however, there are no randomized clinical trials comparing surgery to CTRT in IB3 patients. Because of this, the optimal management of IB3 remains controversial [12]. The GOG 92 trial compared adjuvant RT vs. observation following radical hysterectomy (RH) in IB patients with intermediate risk factors. The results showed an improved disease-free survival (DFS) rate in the adjuvant radiotherapy group [13]. A subsequent update from the same study, with a longer follow-up, reported no benefit to overall survival [14].

Recent ESGO/ESTRO/ESP guidelines [4,15,16,17] indicate adjuvant chemoradiation after RH in patients with high risk factors for recurrence (lymph node involvement, parametrial upstaging, or positive surgical margins). In patients with intermediate risk factors (two out of the following: one-third deep stromal invasion, capillary-like space involvement, or a tumor size > 4 cm), adjuvant radiotherapy is optional [18]. In the case of adequate surgeries performed by experienced teams, observation is considered a valid alternative [19]. These findings have reignited an interest in radical surgery for stage IB3 cervical cancer.

In this study, we propose a laparoscopic nerve-sparing type C2 radical hysterectomy (nsLRH) based on the Querleu–Morrow classification [20] to treat IB3 cervical cancer without adjuvant treatment in the presence of intermediate risk factors. We report on the surgical technique with a critical appraisal of the surgical anatomy. The aim is to investigate the safety, efficacy and oncological outcomes of nsLRHs.

## 2. Materials and Methods

### 2.1. Study Design and Population

Between 2009 and 2023, a prospective observational study was conducted on patients with histology confirmed cervical cancer >4 cm in size, initially staged as IB2 [21], later denominated IB3 as per the revised FIGO classification, to undergo a nsLRH; the follow-up of the patients continued until the end of 2023. For the sake of clarity, the current FIGO classification [1] was used for the study and patients were defined as IB3, although many patients were treated before 2018. The study was approved by the Oxford University Hospital Trust (IRB 3266). The institutions involved were the Fondazione San Raffaele (Cefalù, Italy), Oxford University Hospital (Oxford, UK) and Padua University Hospital (Padua, Italy).

The inclusion criteria were a proven histology of squamous cell carcinoma (SCC) or adenocarcinoma of the cervix; FIGO stage IB3 (invasive carcinoma confined to cervix, ≥4 cm at its greatest dimension); and 18 years of age or above. The exclusion criteria were lymph-nodes involved at the frozen section, disease beyond stage IB3, a histology other than SCC or adenocarcinoma, and comorbidities precluding radical surgery. A departmental Cervical Cancer Database was used to record, monitor and audit all outcomes of interest.

### 2.2. Treatment and Follow-Up

During the study period, all consecutive patients meeting inclusion criteria were elected to a stepwise triage process as follows: a clinical examination, magnetic resonance imaging (MRI), positron emission tomography (PET) scan, and a multi-disciplinary team (MDT) meeting. Patients with evidence of disease spread beyond the cervix during this selection process were referred for definitive CTRT with external beam and brachytherapy. All patients were given explanations and offered the option of surgery or definitive CTRT. Triaged on the basis of the above clinical and radiological staging, patients underwent examination under anesthetic, laparoscopic bilateral pelvic lymphadenectomy with frozen section analysis and, if this was negative, a nsLRH was performed. Patients with pT1b3N0M0 upon their final histology were offered a consultation on the merits and risks of observation vs. adjuvant therapy [14]. Patients who were identified as having a final histology with a stage beyond IB3 or exhibiting high risk factors for recurrence [4] were recommended to undergo adjuvant treatment. A dedicated gynecologic oncology pathology team reported on the margin status, lympho-vascular space invasion (LVSI), depth of invasion, and length of the parametrium/paracolpium. Tumor and parametrial size were measured on the paraffin-fixed specimens. The size was measured along the longest axis of the parametrium on the unstretched specimen from the lateral walls of the cervix. From the histopathology, we also recorded the presence of intermediate and high-risk factors for recurrence. All patients underwent a rigorous follow-up program every three months for the first two years post-surgery, every four months for the subsequent two years, every six months in the fifth years, and then annually.

### 2.3. The Surgical Technique

The operation was modified from a previously published technique [22]. The laparoscopy was accomplished with 3 × 5 mm and 2 × 10 mm non-disposable trocars (Karl Storz, Tuttlingen, Germany). The operation started with a bilateral pelvic and parametric lymphadenectomy. The lymphadenectomy technique was standardized over the years in Jena, Germany, and the results were published [22]. Here, only 3 additional steps are reported, incorporating the principles of the Querleu–Morrow classification [20] and of the TMMR [23], which are preparatory to display the anatomic structures to be preserved or resected during the nsLRH.

Firstly, the obturator fossa dissection continues with the removal of the lymph nodes placed dorsal to the obturator plane, leaving behind only the obturator vessels and the nerve (Figure S1). By accessing the arcus tendineus and the plane of the levator ani muscle where the paracolpium and parametrium attach to, the obturator, pubococcygeus and iliococcygeus muscles are exposed. Next is the full skeletonization of the uterine vessels by removal of the lymph nodes located on the uterine artery and veins, with separation of the artery and veins until the middle rectal artery and/or the deep uterine vein are exposed (Figure S2). The last step is the lymphadenectomy of the lumbosacral fossa, removing the lymph nodes dorsal to the external iliac vessels medial to the psoas muscle and above the lumbo-sacral trunk and sciatic nerve which are exposed (Figure S3). The purpose of this step is to free the upper part of the pelvic sidewall. The pelvic lymphadenectomy is performed with the “en-bloc” technique, respecting the integrity of the lymph nodes, which are not manipulated, and are only extracted using disposable endo-bags (Karl Storz, Tuttlingen, Germany) to avoid the risk of contamination. The endo-bags are removed through a 10 mm subcostal port to minimize the risk of spillage. If the lymph nodes are tumor-free at the frozen section, the nsLRH is performed.

No resection is performed until the field is fully displayed, which includes the isolation and sparing of the nerves, dissection of the ureter and complete exposure of the parametrium and paracolpium. The first part of the surgery aims to spare the autonomic nerves (hypogastric and splanchnic) and the ureter. We identify the hypogastric nerves at the level of the sacral promontory where they run lateral to the meso-sigmoid, and then in the broad ligament dorsal to the ureter and ventral to the parametrium. On each side, the nerve is followed until it meets the splanchnic pelvic nerves originating from the sacral roots and forms the inferior hypogastric plexus (Figure S4). The nerve plane is dorsal to the plane of the uterine vessels and is roofed by the deep uterine vein. Therefore, the uterine vessels are coagulated and cut at their origin from the internal iliac artery. In some cases, a vessel loop is passed around the nerve bundle to facilitate the dissection from the parametrium. The distal part of the nerve bundle is dissected concomitantly with the dissection of the bladder pillar.

The next step is the isolation of the ureter from the pelvic brim to the bladder pillar to separate it from the parametrium. This is achieved by gentle traction, preserving a rim of meso-ureter where it crosses the uterine vessels. At this point, the ureter is more tenaciously adhered to the parametrium. Therefore, the uterine vessels, previously coagulated at their origin from the internal iliac artery, are used for traction to pinpoint the ureter tunnel and the parametrium. To fully expose the parametrium and later the paracolpium, the ureter must be released ventrally and dorsally. This will also facilitate the sparing of the distal nerve bundle, particularly in the bladder pillar, which is divided into a supra- and infra-ureteral part. The latter division is made by the ureter crossing the bladder pillar. We believe that the infra-ureteral part of the bladder pillar, which carries important blood supply to the ureter, can be safely spared without compromising the radicality of the procedure. However, the supra-ureteral part of the bladder pillar holds the distal part of the ureter bond to the parametrial structures. Therefore, it needs to be dissected to push away the most distal portion of the ureter and fully expose the vagina and the dorsal part of the paracolpium.

Once the ureter and nerves are dissected and spared, the parametrium/paracolpium is fully exposed. The rectum is mobilized leaving the rectal pillar exposed. No uterine manipulator is used. The parametrial structures are particularly well-exposed by the traction of the uterus contralateral to the side of the resection. Such retraction is applied with a long instrument placed in the subcostal port pulling the uterus. The nerves and ureter are pulled ventrally, making the dissection plane clear. At this stage, through bipolar scissors coagulation and cutting, the fully dissected parametria/paracolpia are resected.

Lastly, once the vagina is fully exposed and cleared from the bladder and the rectum, the colpotomy is performed with monopolar coagulation. To prevent the risk of contamination, the vagina is opened anteriorly and posteriorly about three cm so that the cuff can be sealed with the tenacula introduced through the vagina. Alternatively, a laparoscopic loop suture is applied around the distal vagina, before the colpotomy, to isolate the specimen from the peritoneal cavity. The specimen is extracted through the vagina, which is then closed with an intra-corporeal suture. In patients with positive lymph nodes at the frozen section, the surgery is abandoned, and patients are referred for chemoradiation.

### 2.4. Endpoints of the Study

The study’s endpoints were the efficacy, safety and survival outcomes of nsLRHs. Efficacy was measured by the rate of complete resection (CR), defined as 10 mm tumor-free vaginal and parametrial margins, and safety was defined by the morbidity graded with the Clavien–Dindo classification [24]. The survival outcomes are based on the DFS and OS rates. DFS was defined as the time interval between the date of surgery and the documented first recurrence or the last follow-up, while OS was defined as the time interval between the date of surgery and death or last follow-up.

### 2.5. Statistical Analysis

The data were analyzed using Fisher’s exact test for the categorical variables and Student’s t-test for the continuous variables. The survival data were projected with the Kaplan–Meier method; the log-rank testing was used as a comparison (for descriptive purposes). A result of *p* < 0.05 was considered statistically significant. The sample size was calculated with an optimal two-stage design [25]. The study was designed to aim for a high expectation of efficacy and safety. Efficacy was defined as the rate of CR and was set at 0.85 (p1), considering 0.70 an insufficient level of efficacy (p0). Safety was measured by the number of patients experiencing a recurrence within 6 months of the surgery. We used the survival data reported in 3 major clinical trials [3,4,13] as a reference.

A minimum of 45 patients were required to show a safety of 0.85 and an efficacy rate of 0.90, with a type I error of 0.05 and a power of 90%. The first stage was meant to enroll 24 patients, with a probability for early termination of 0.65, and was sanctioned if more than 3 patients experienced a recurrence within the first 12 months. An additional study discontinuation rule was set at 24 months if 15% of the patients experienced a recurrence. Since this was not a randomized trial and considering the small numbers, we decided to undertake a “per protocol” survival analysis, which closed when 75% of the patients had at least 60 months of follow-up or experienced a recurrence.

## 3. Results

### 3.1. Patients’ General Features and Tumor Characteristics (Table 1)

During the study period, a total of 74 patients with IB3 FIGO stage cervical cancer underwent laparoscopic pelvic lymphadenectomy. Six patients (8.1%) had tumor-involved lymph nodes at the frozen section analysis (N+) and were referred for definitive chemoradiotherapy (CTRT). The remaining 68 patients (91.9%) had negative lymph nodes at the frozen section and underwent a nsLRH.

**Table 1:** Patient and tumor characteristics.

	All Patients (*n* = 74)
Age, median (range)	46 (20–77)
Pre-menopausal status, *n* (%)	35 (47.3%)
Previous malignancies, *n* (%)	0
BMI, median (range)	29 (22–35)
Histology, *n* (%)	
Squamous	50 (67.6)
Adenocarcinoma	24 (32.4)
Grade, *n* (%)	
G1	19 (25.7%)
G2	29 (39.2%)
G3	26 (35.1%)
Presurgical FIGO Stage	
IB3	74 (100%)

### 3.2. Surgical Outcomes (Table 2)

No conversion to laparotomy occurred. The median surgery time was 236 min (range between 96 and 669 min) and the median estimated blood loss was 95 cc (range between 15 and 300 cc). No obvious intra-operative complication occurred. The indwelling urinary catheter was removed two days after the procedure (range between 2 and 4 days). The median hospitalization stay was three days (range between 2 and 13 days).

A complete resection with negative margins was achieved in all patients, including three patients with microscopic parametrial involvement, with margin clearances of 28 mm, 26 mm and 15 mm. The median tumor size was 58 mm (range between 40 and 87 mm). Figure S5 shows a typical nsLRH specimen.

**Table 2:** The surgical/pathologic outcomes of patients who underwent a nsLRH (*n* = 68).

	Surgical Patients (*n* = 68)
Conversion to laparotomy	-
Operative time, median (range)	236 (96–699)
EBL, median (range)	95 (15–300)
Hospital days, median (range)	3 (2–13)
Tumor size (mm), median (range)	58 (40–87)
Parametrial length (mm), median (range)	
Left	48 (30–55)
Right	46 (34–61)
Pelvic lymph nodes, mean (range)	
Left	15.8 (12–27)
Right	18.9 (13–37)
Tumor-involved lymph nodes, *n* (%)	7 (10.3%)
TNM staging (FIGO stage)	
pT1b3pN0cM0 (IB3)	57 (83.8)
pT2a2pN0cM0 (IIA2)	1 (1.5%)
pT2bpN0cM0 (IIB)	3 (4.4%)
pT1b3pN1cM0 (IIIC1)	2 (2.9%)
pT2a2pN1cM0 (IIIC1)	4 (5.9%)
pT2bpN1cM0 (IIIC1)	1 (1.5%)

EBL: Estimated blood loss; LVSI: lympho-vascular space invasion; N+: positive lymph node/s; R1: residual disease left.

### 3.3. Study Groups and Outcomes

**Figure 1:**
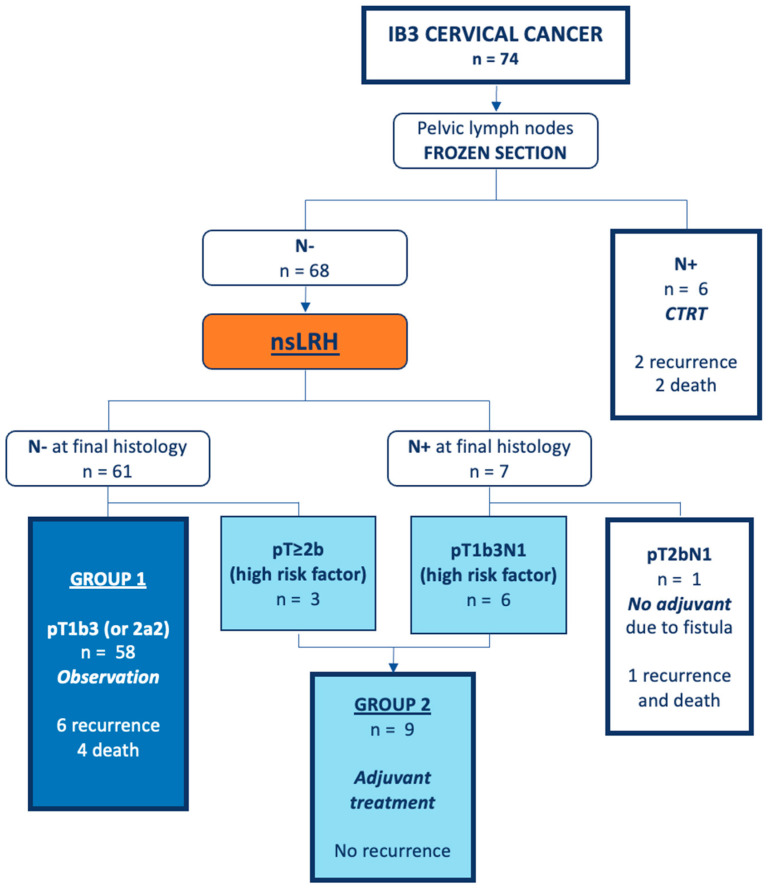
Study flow chart. IB3, FIGO stage 2009 IB3; N+, positive lymph nodes; N−, negative lymph nodes; CTRT, chemoradiation therapy; and nsLRH, nerve-sparing laparoscopic radical hysterectomy.

Fifty-eight patients underwent a nsLRH without adjuvant treatment. They form the study “per protocol” group (Group 1); all of these patients presented at least two of three intermediate risk factors (>one-third stromal invasion, capillary lymphatic space involvement and/or large clinical tumor diameter) [9,10]. Ten patients had a stage change or high-risk factors (positive pelvic lymph nodes and/or microscopic involvement of the parametrium) [4] found at their final histology (Group 2). Specifically, two patients had one and two involved lymph nodes, respectively (pT1b3pN1cM0 or FIGO IIIC1), three patients had microscopic parametrial involvement (pT2bpN0cM0, FIGO IIB) and four had vaginal and lymph nodal involvement (pT2a2pN1cM0 or FIGO IIIC1). All of these patients underwent adjuvant radiotherapy. One patient had microscopic parametrial involvement and one positive lymph node (pT2bpN1cM0 or FIGO IIIC1), but had no adjuvant radiotherapy due to a uretero-vaginal fistula. Figure 1 reports the study flow chart.

**Figure 2:**
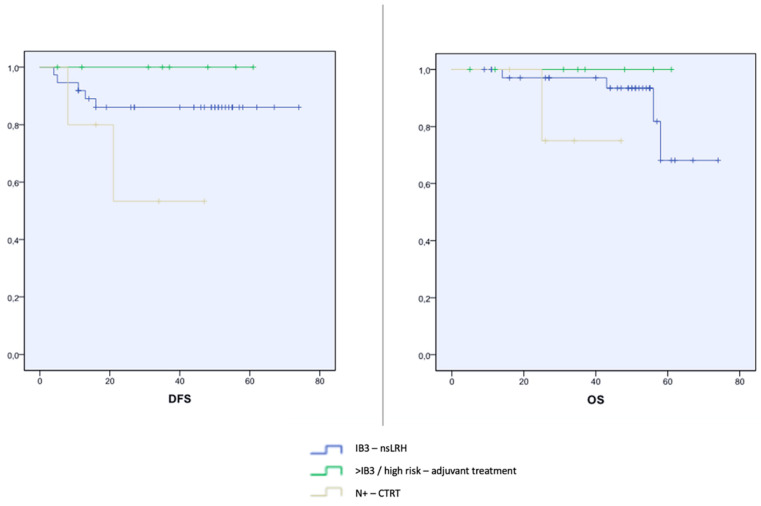
Kaplan–Meier curves. IB3, FIGO stage 2009 IB3; N+, positive lymph nodes; N−, negative lymph nodes; CTRT, chemoradiation therapy; and nsLRH, nerve-sparing laparoscopic radical hysterectomy.

Six patients with nodal involvement (N+) at frozen section had definitive CTRT; of those, two patients experienced a recurrence and died of disease at 53 months median follow-up (range between 37 and168 months). DFS and OS rates in N+ patients were both 66%. In Group 1, at 68 months median follow-up (range 12–168 months), six patients had a recurrence and four died of disease. In Group 2, despite upstaging, at 64 months median follow-up (range 18–147), no recurrence was recorded. The Kaplan–Meier curves are reported in Figure 2. At a 68 months median follow-up, DFS rate was 89.7% in Group 1 and 100% in Group 2. The overall survival (OS) rate was 93.1% and 100%, respectively.

### 3.4. Post-Operative Complications (Table 3)

In Group 1, no grade 4–5 complications occurred. The grade 3 complication rate was 8.8%. Notably, three patients in Group 1 and one in Group 2 experienced a uretero-vaginal fistula. The CT urogram showed a leak of contrast in the peritoneal cavity and drainage through the vagina from a small fistula on the most distal part of the ureter. They had ureteric stents placed to attempt a conservative management. A repeated scan at 6–8 weeks showed complete healing in three patients and the stents were removed. In one patient, a ureteral reimplant was successfully performed. Another patient, shortly after the nsLRH, complained of severe leg pain. A diagnosis of compartment syndrome was promptly made and a fasciotomy resolved the symptoms with no long-term morbidity. The Grade 1 and 2 complication rates were 11.7% and 2.9%, respectively; in five patients, a bladder dysfunction (lack of sensation in three patients and retention in two) exceeding 8 weeks was reported. All patients had the urinary dysfunction sorted within 60 days, except for one patient who had long-term self-catheterization. It was a large tumor, 71 mm, which made the nerve-sparing procedure challenging due to the proximity of the tumor and an attempt to not compromise radicality.

**Table 3:** Intra-/post-operative complications (Clavien–Dindo classification) [24].

	All Patients (*n* = 68)
Intra-operative complications, *n* (%)	-
Post-operative complications, *n* (%)	16 (23.5%)
**Grade I**	8 (11.7%)
Urinary dysfunction	6
Lymph cyst	2
**Grade II**	2 (2.9%)
Urinary infection	1
Lymphoedema	1
**Grade III**	6 (8.8%)
Uretero-vaginal fistula	4
Compartment syndrome	1
Hematoma	1
**Grade IV**	-

## 4. Discussion

The treatment of IB3 FIGO stage (2018) cervical cancer remains an area of debate. No randomized trial has yet compared primary surgery to CTRT. Thus, both methods are now considered as valid therapeutic options [19]. Few retrospective studies have compared RH vs. CTRT in IB3 patients, reporting an interestingly higher OS rate in patients treated with RH (76.4% vs. 94.0%, 73.3% vs. 83.2% and 72.5% vs. 81.5%, respectively [26,27,28]). In addition, a recent Cochrane systematic review stated that there is no evidence for or against radical hysterectomy eventually followed by concurrent CTRT versus CTRT alone for these patients [29]. Therefore, in anticipation of further studies, treatment-related morbidity can be a decisive factor. The survival outcomes of our study are encouraging. Compared to historical studies including similar patients, with the limitation of a different sample size, DFS and OS rates in our study are higher than the ones recorded in the GOG 92 surgery alone arm (DFS of 72% and OS of 79%) and very similar to adjuvant radiotherapy arm (DFS 85% and OS 87%). In addition, the survival outcomes are higher than those in the GOG 123 trial (DFS of 79% and OS of 83%), with a lower overall morbidity rate [6].

The current trend is to avoid dual-modality treatment to reduce morbidity [9]. In the GOG 92 trial, the patients with intermediate risk factors needed adjuvant radiotherapy after surgery to improve the DFS rate by 13%, although no statistical improvement was registered for OS [13,14]. The perception of this trial in the gynecologic oncology community was that surgery alone led to inferior results than surgery and adjuvant radiotherapy. Notwithstanding the lack of a randomized clinical trial, definitive CTRT became the preferred treatment, and radical surgery the alternative [10,30]. However, it should be noted that radical CTRT in IB3 patients resulted in 48% of persistent disease [31]. That implies a significant risk of dual treatment. In addition, tumor size was identified as a factor of poor complete pathological response [32,33], as was recently confirmed by the results of the EMBRACE I study [34]. The most common post-chemoradiation treatment is surgery, which is more challenging and associated with a higher morbidity when performed after a previous treatment. In addition, surgery in the form of an extra-fascial hysterectomy failed to prove any significant advantage [10]. Finally, of note is the scenario where patients whose response to external beam radiotherapy is insufficient to permit brachytherapy ending with a sub-optimal radiotherapy dose. Again, initial tumor volume is the strongest predictor of response to external beam radiotherapy [32,33,34,35]

Considering its similar survival outcomes, reducing the toxicity of treatment rate remains an important outcome for IB3 stage cervical cancer. The largest study with a prospective morbidity assessment after CTRT, the EMBRACE I study reported estimates of severe (grade 3–4) late gastrointestinal (GI), genitourinary, vaginal and fistula events after 5 years of 8.5%, 6.8%, 5.7% and 3.2%, respectively [33,34,36,37,38]. The long-term outcomes of the GOG 123 trial, which demonstrate the superiority of exclusive CTRT over RT in IB3 tumors, reports a 34.9% G3-G4 overall morbidity rate, mainly from transient hematologic and GI events [6].

In the current study, a nsLRH led to a complete resection (10 mm free) in all patients, with no intra-operative morbidity, while post-operative morbidity was significantly lower than what is reported in chemoradiation trials [39]. The most notable post-surgical event recorded was a ureteric fistula, which manifested in all patients 2–3 weeks after the procedure, supporting the concept of ischemic damage to the ureter. Notably, no direct ureteral injury occurred during the procedures. In the last 38 patients, after a change in the procedure, as already mentioned, no fistula occurred, possibly due to the systematic sparing of the infra-ureteral part of the bladder pillar. However, the size of the tumor and the attempt to achieve free margins demand a significant dissection. A recent meta-analysis highlighted that patients undergoing radiotherapy face higher risks of long-term bladder and bowel dysfunction as well as sexual dysfunction and psychosocial consequences, including dyspareunia, vaginal stenosis, shortening, dryness, fibrosis and urinary incontinence, especially among younger patients [36,40]. In addition, these symptoms are long-lasting and sometimes permanent [41]. Another instance is the risk of a second malignancy following radical radiotherapy [42] since patients with cervical cancer are, on average, young and with a long-life expectancy. Finally, a cost-effectiveness analysis comparing surgery vs. chemoradiation for IB3 clearly demonstrates a cost savings with primary surgery, even when accounting for the complications of surgery [43,44].

With regard to the surgical technique, nsLRH is a type of surgery that adapts to a large tumor size whereby no compromise is made on the radicality. The dissection is tailored to extend in laterality, not necessarily in depth. The latter is, in IB3 patients, the only way to spare the splanchnic and pelvic nerves, which are literally untangled from the parametrial structures. A nsLRH does not exactly fit in the classes reported in the Querleu–Morrow classification [20]; it has the radicality of C2, but it also includes nerve sparing, as is suggested by other authors [45,46,47]. Whether the length of the parametrium matters in terms of survival has yet to be demonstrated. However, all studies reporting a considerable effort on the radicality of the surgery consistently display an improved survival outcome [22,45]. Our study was conducted as a feasibility study testing the toxicity and the efficacy of the surgery. The non-randomized setting and the number of patients clearly limit the value of the results. However, the encouraging outcomes in toxicity and survival, support the idea that surgery is a valid option at the IB3 stage.

Recently, the LACC trial challenged the use of a minimally invasive technique in patients with cervical cancer [48]. However, it was published after this study was conducted. Additionally, it did not include IB3 patients, which was the focus of our study, and a tumor size greater than 4 cm was specifically mentioned as an exclusion criterion. While we respect the efforts and the results of the LACC trial, it must be noted that randomization did not prevent a significant procedural bias between the groups, namely, the use of a manipulator in the minimally invasive group. As described in a recent study [49] which had a large population and yielded a higher survival rate, by squeezing an active cancer and opening the vagina right on top of the area of pressure, the manipulator poses a serious risk of spread, spillage and contamination. In addition, it cannot be excluded that the increased pressure may induce spread in the lymphatic and blood streams. For clarity, in the LACC trial, there were no details and strict criteria on the colpotomy, the handling of lymph nodes or directives on how to avoid contamination between the cancer and the surgical field, showing significant liberality in the surgical technique (closure of the vagina, extraction of the lymph nodes) and surgical expertise (only ten procedures were needed to enter the study). While surgical oncological “hygiene” is relevant for all types of surgery, it becomes essential when considering the potential risk for tumor spread by circulating CO_2_. Indeed, the authors recognize in the manuscript the potential risk associated with the manipulator, but they state in a later letter that such risk is speculative [50]. To emphasize this, a few later studies disagreed with the LACC trial [49,50,51]. The SUCCOR study confirmed that the use of a uterine manipulator carries a worse prognosis, while no such risk was identified without it [52]. The MEMORY study confirmed comparable outcomes in terms of OS and DFS rates if a laparoscopic radical hysterectomy was performed by an experienced gynecologic oncologist [53]. In our opinion, subsequent studies will have to avoid the use of uterine manipulators to protect the safety of their patients and create the correct conditions for comparison. Currently, there are four ongoing randomized trials on this topic [54,55,56,57], one of which specifically focuses on the use of minimally invasive surgery in IB3 cases [55]; evaluating their outcomes will be crucial in determining the most effective and safe treatment for these patients.

## 5. Conclusions

The results of our study support the potential use of a nsLRH as a safe and effective treatment for IB3 FIGO stage cervical cancer. The findings demonstrate promising oncological outcomes with minimal intra-operative morbidity and lower post-operative complications compared to chemoradiation. Despite the ongoing debate, our study supports the role of surgery in this specific patient group. Continued refinement of surgical techniques and adherence to oncological principles remain crucial for enhancing outcomes in IB3 cervical cancer patients.

## Data Availability

The data presented in this study are available upon request from the authors.

## Supplementary Materials

The following supporting information can be downloaded at: https://www.mdpi.com/article/10.3390/cancers16193355/s1, Figure S1: Pelvic lymphadenectomy: final situs, right side; Figure S2: Isolation of the left uterine vessels during the parametric lymphadenectomy; Figure S3: Left lumbo-sacral trunk lymphadenectomy: final situs; Figure S4: The splanchnic pelvic nerves; Figure S5: nsLRH specimen with parametrial measurement.
