# Supplementary material for: Nerve-Sparing Laparoscopic Radical Hysterectomy (nsLRH) without Adjuvant Therapy in FIGO Stage IB3 Cervical Cancer Patients: Surgical Technique and Survival Outcomes
Source: Cancers (Basel). 2024 Sep 30;16(19):3355. doi: 10.3390/cancers16193355 (PMC11475574; doi:10.3390/cancers16193355)
Supplement: Supplementary file 1 [file cancers-16-03355-s001.zip › cancers-3188900-supplementary.pdf]

IB3 supplementary materials

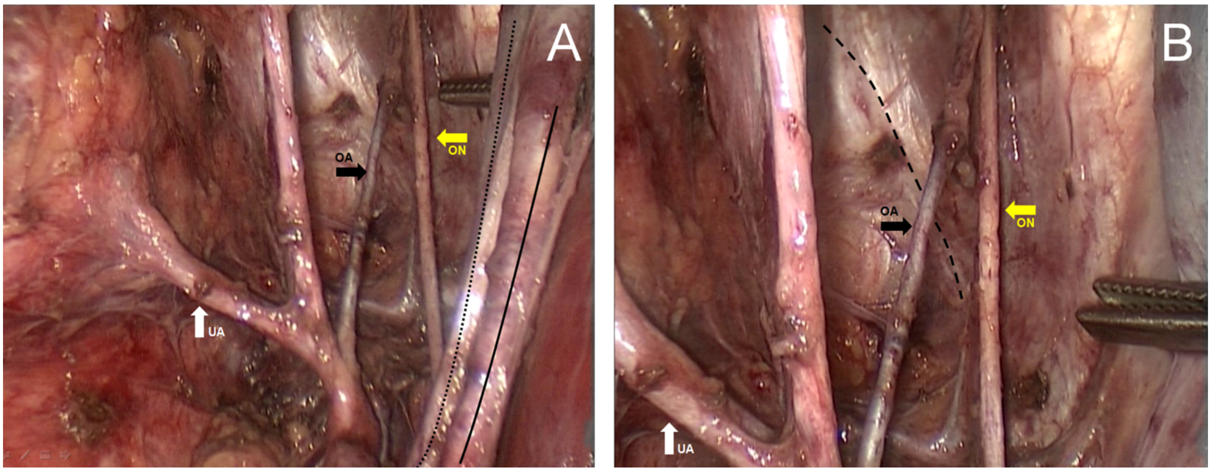

Figure S1. A-B Pelvic lymphadenectomy: final situs; right side. Legend: Fig 1A. Yellow arrow: obturator nerve (ON); black arrow: obturator artery (OA); white arrow: uterine artery (UA); black dotted line: external iliac vein; black line: external iliac artery. Fig 1B. yellow arrow: obturator nerve (ON); black arrow: obturator artery (OA); white arrow: uterine artery (UA); black dotted line: arcus tendineum. (print in color)

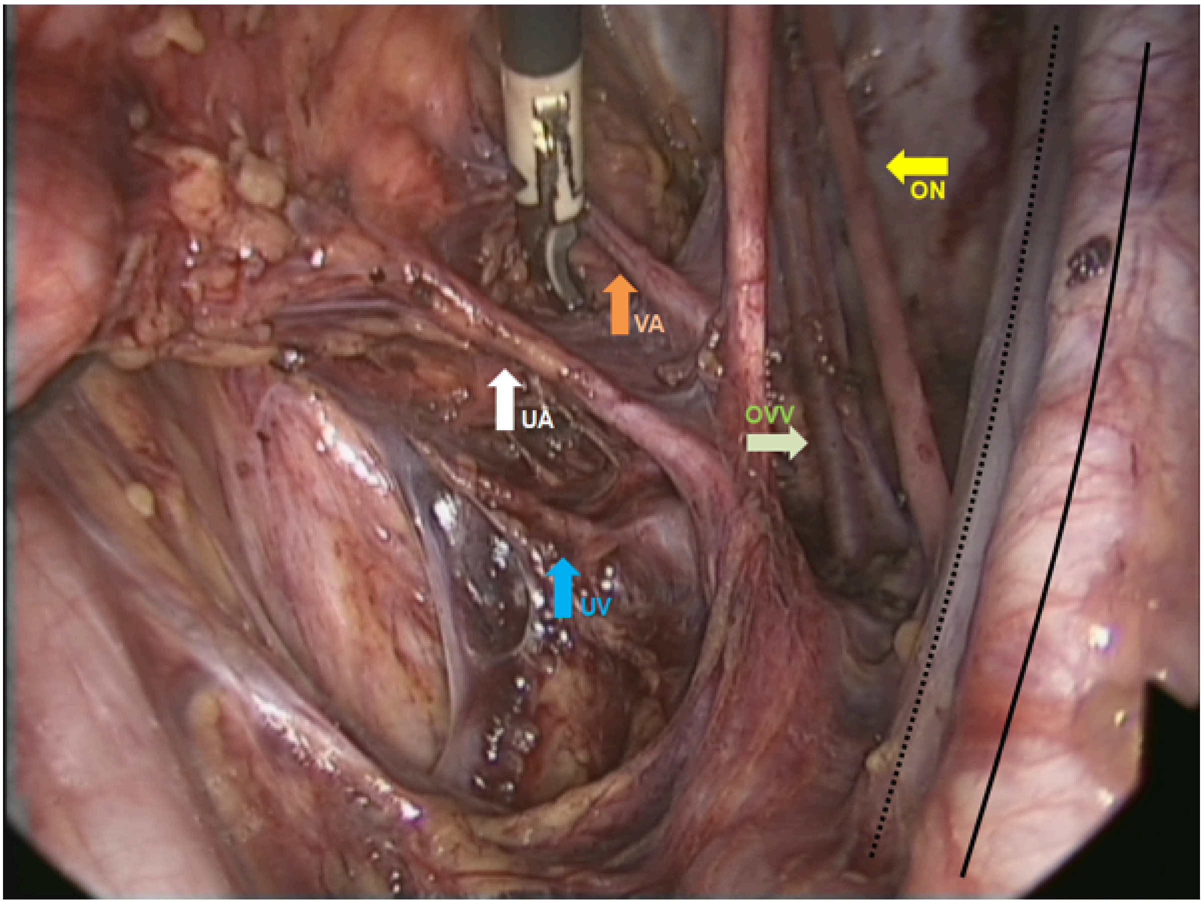

Figure S2 Isolation of the left uterine vessels during the parametric lymphadenectomy. Legend: Yellow arrow: obturator nerve (ON); green arrow: obturator vessels (OVV); white arrow: uterine

artery (UA); blue arrow: deep uterine vein (DUV); orange arrow (VA): vaginal artery; black dotted line: external iliac vein; black line: external iliac artery. (print in color)

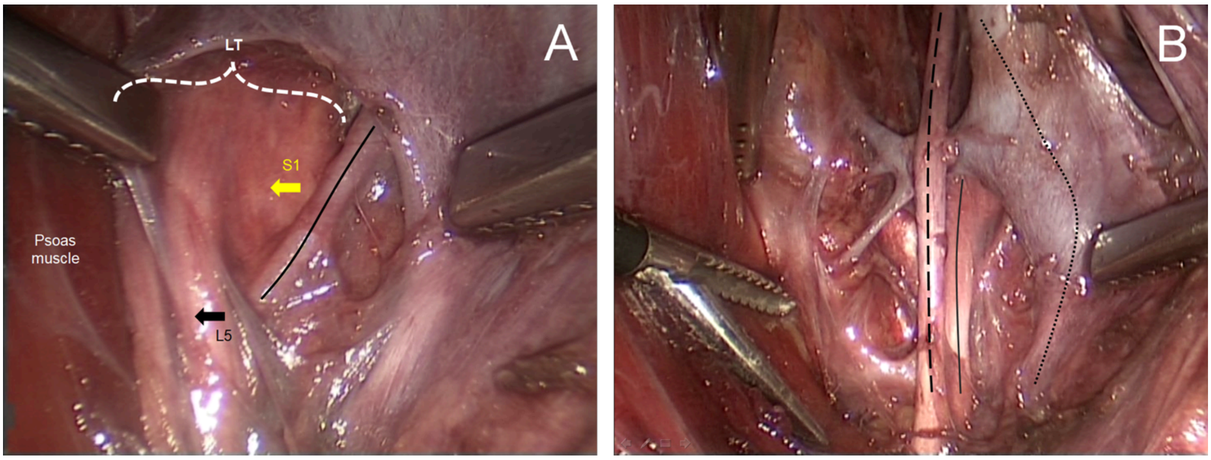

Figure S3. A-B Left lumbo-sacral trunk lymphadenectomy: final situs. Legend: Fig 3A. Black arrow: lumbar root (L5); yellow arrow: sacral root (S1); dotted white: lumbosacral trunk (LT); black line: gluteal artery. Fig 3B. Dotted line: external iliac vein; black line: lumbosacral trunk; dashed line: obturator nerve. (print in color)

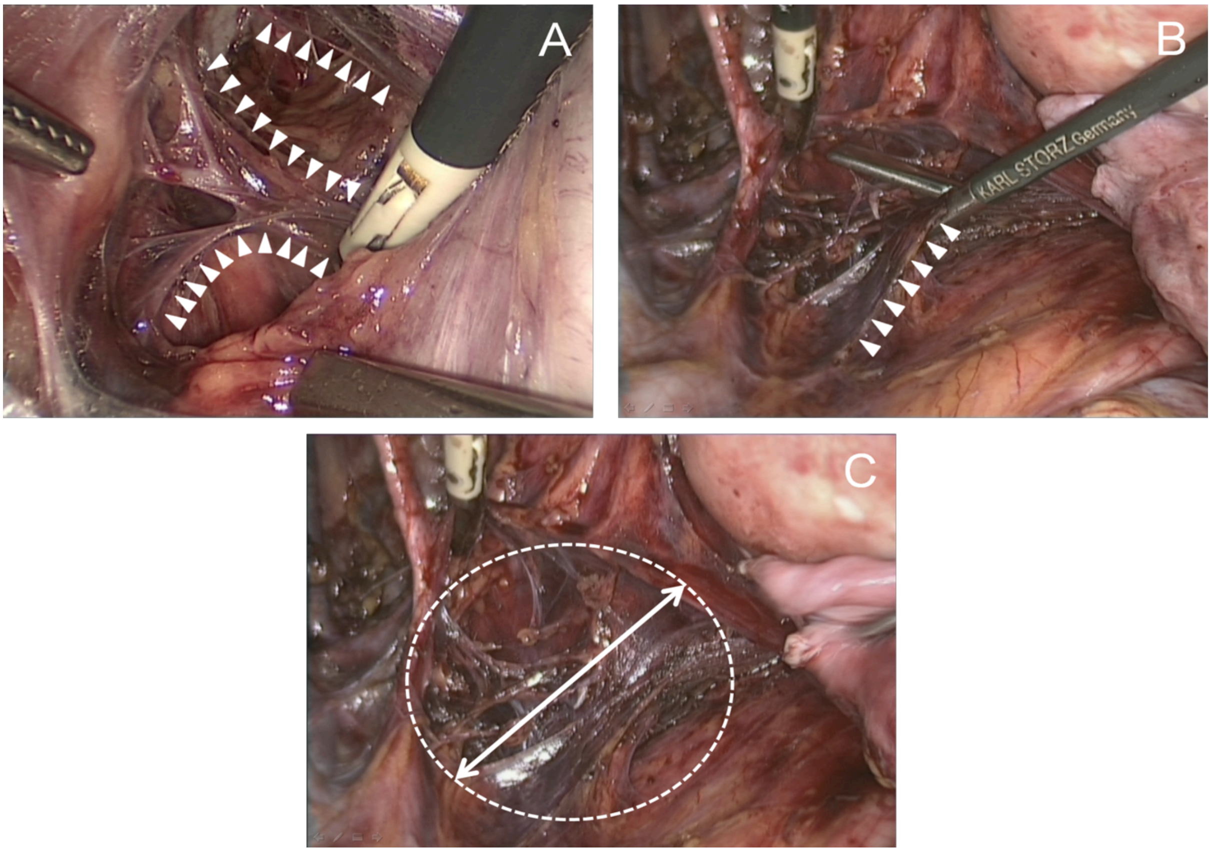

Figure S4. The splanchnic pelvic nerves (A), the inferior hypogastric plexus (B) and the pelvic plexus (C). (print in color)

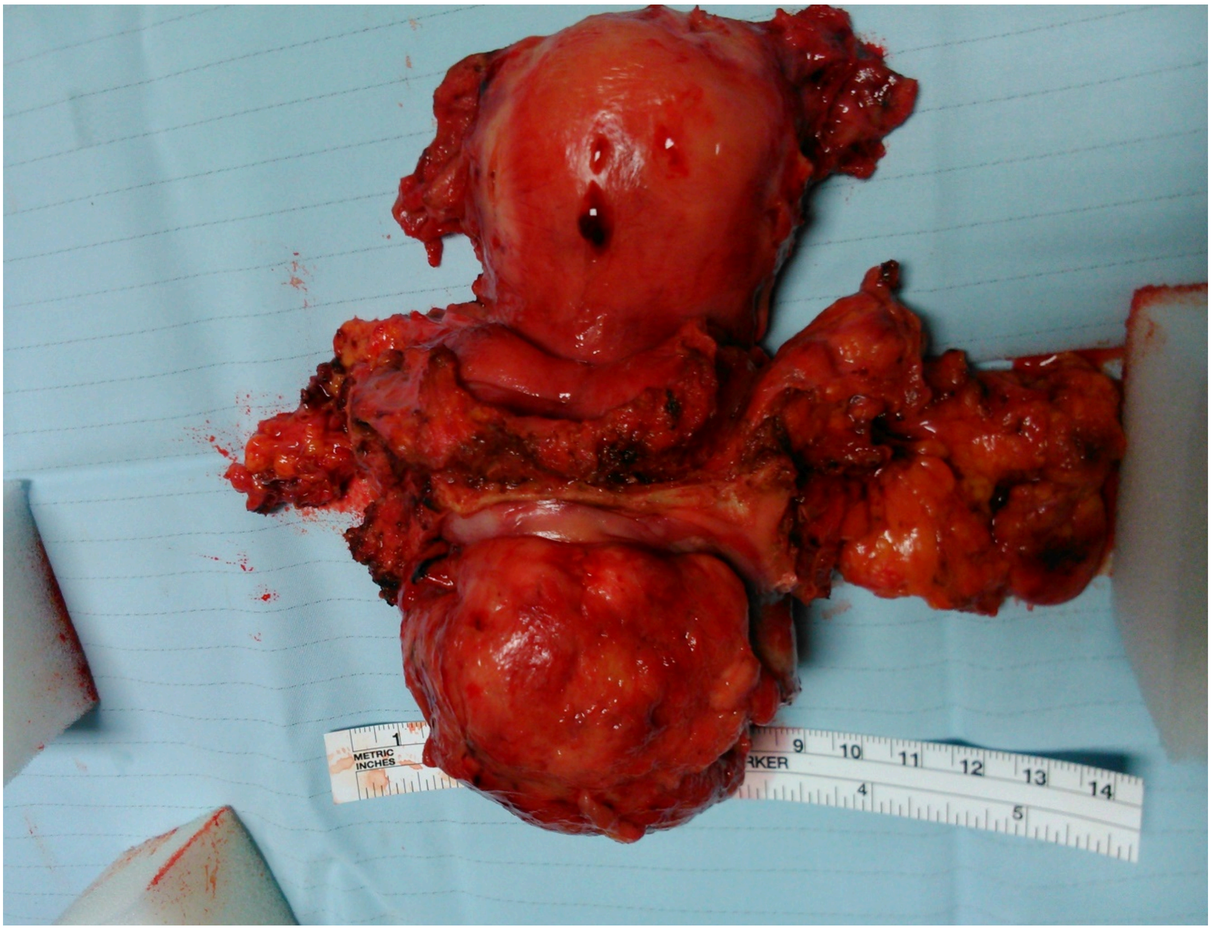

Figure S5: nsLRH specimen with parametrial measurement. (print in color)
